# Individual differences in beta-band oscillations predict motor-inhibitory control

**DOI:** 10.3389/fnins.2023.1131862

**Published:** 2023-03-01

**Authors:** Qian Ding, Tuo Lin, Guiyuan Cai, Zitong Ou, Shantong Yao, Hongxiang Zhu, Yue Lan

**Affiliations:** ^1^Department of Rehabilitation Medicine, Guangzhou First People’s Hospital, South China University of Technology, Guangzhou, Guangdong, China; ^2^Department of Rehabilitation Medicine, Guangdong Provincial People’s Hospital (Guangdong Academy of Medical Sciences), Southern Medical University, Guangzhou, Guangdong, China; ^3^Guangzhou Key Laboratory of Aging Frailty and Neurorehabilitation, Guangzhou, Guangdong, China

**Keywords:** EEG, motor inhibitory control, beta-band oscillations, functional connectivity, global efficiency

## Abstract

**Objective:**

The ability of motor-inhibitory control is critical in daily life. The physiological mechanisms underlying motor inhibitory control deficits remain to be elucidated. Beta band oscillations have been suggested to be related to motor performance, but whether they relate to motor-inhibitory control remains unclear. This study is aimed at systematically investigating the relationship between beta band oscillations and motor-inhibitory control to determine whether beta band oscillations were related to the ability of motor-inhibitory control.

**Methods:**

We studied 30 healthy young adults (age: 21.6 ± 1.5 years). Stop-signal reaction time (SSRT) was derived from stop signal task, indicating the ability of motor-inhibitory control. Resting-state electroencephalography (EEG) was recorded for 12 min. Beta band power and functional connectivity (including global efficiency) were calculated. Correlations between beta band oscillations and SSRT were performed.

**Results:**

Beta band EEG power in left and right motor cortex (MC), right somatosensory cortex (SC), and right inferior frontal cortex (IFC) was positively correlated with SSRT (*P*’s = 0.031, 0.021, 0.045, and 0.015, respectively). Beta band coherence between bilateral MC, SC, and IFC was also positively correlated with SSRT (*P*’s < 0.05). Beta band global efficiency was positively correlated with SSRT (*P* = 0.01).

**Conclusion:**

This is the first study to investigate the relationship between resting-state cortical beta oscillations and response inhibition. Our findings revealed that individuals with better ability of motor inhibitory control tend to have less cortical beta band power and functional connectivity. This study has clinical significance on the underlying mechanisms of motor inhibitory control deficits.

## 1. Introduction

Motor-inhibitory control refers to the ability that inhibits inappropriate motor responses and expresses more appropriate responses, which is considered as an important ability in daily life (Chowdhury et al., 2017). The ability of motor-inhibitory control can be measured by stop signal task (SST) (Aron, 2011; Bari and Robbins, 2013; Schall et al., 2017), in which participants are instructed to inhibit an already initiated action. Stop-signal reaction time (SSRT) can be estimated based on the latency to inhibit a prepotent response (i.e., stopping efficiency). Prolonged SSRT suggests poor ability of motor-inhibitory control (Chowdhury et al., 2017). It has been reported that SSRT tend to be prolonged in conditions such as Parkinson’s disease (PD) (Jenkinson and Brown, 2011), attention deficit/hyperactivity disorder (Lijffijt et al., 2005) and schizophrenia (Badcock et al., 2002). The physiological mechanisms underlying the motor inhibitory control deficits remain less clear (Bari and Robbins, 2013).

As a common neuroimaging approach, electroencephalography (EEG) has been widely applied in the field of neurophysiological research. Ongoing spontaneous EEG oscillations are usually categorized into five frequency bands, including delta, theta, alpha, beta, and gamma bands. In particular, rhythmic activity in the beta band (i.e., 15–30 Hz) is classically considered as being related to sensorimotor functions (Pfurtscheller et al., 1996), but the functional role of beta-band activity has not been fully elucidated. Beta-band activity (i.e., EEG power) has been suggested as a signature of an active process that promotes the existing motor set whilst compromising neuronal processing of new movements (specifically related to maintain the status quo). Excessive beta-band activity has been associated with worse motor performance (Gilbertson et al., 2005; Androulidakis et al., 2006, 2007). Although some studies have reported positive correlations between beta band activity and impulsivity (Threadgill and Gable, 2018; Wendel et al., 2021), whether beta-band activity is related to the ability of motor inhibitory control remains unclear. Based on the theoretical link between motor inhibitory control and impulsivity, we speculate that beta band activity is less in individuals with worse ability of motor inhibitory control.

Functional connectivity stands for the synchrony of cortical activity in anatomically distinct but functionally collaborating brain regions (Vecchio et al., 2019). Unlike EEG power reflecting oscillatory synchronization within local cortical neuronal populations, functional connectivity represents neuronal synchronization between distributed cortical regions (Silberstein et al., 2005). Graph theory analysis is an approach that characterizes functional brain network based on functional connectivity (Park et al., 2014). Global efficiency refers to the average of interregional efficiency between each pair of brain region over the whole brain. As one of the most common metrics in graph theory analysis, global efficiency represents the efficiency in transporting information at a global scale (Park et al., 2014). Some studies investigated the relationship between beta band functional connectivity and motor performance, reporting that individuals with greater beta band functional connectivity tend to have worse motor performance (Gilbertson et al., 2005; Silberstein et al., 2005). However, whether there is a relationship between beta band functional connectivity and motor inhibitory control remains unclear. Investigating the relationship between beta band functional connectivity and global efficiency would provide valuable information on understanding the neural mechanisms of motor inhibitory control deficits.

In present study, we investigated the relationship between beta-band oscillations and motor inhibitory control (i.e., SSRT). We anticipated that beta band power, functional connectivity, and global efficiency would be positively correlated with SSRT. Our findings will have implications on understanding physiological mechanisms of motor inhibitory control deficits and possibly inform the development of new treatment for inhibitory control deficits.

## 2. Materials and methods

### 2.1. Participants

A total of 30 healthy adults [8 males; mean age: 21.6 (SD = 1.5) years] participated in this study. Written informed consent was obtained prior to enrollment. All procedures were approved by the Guangzhou First People’s Hospital Human Research Ethics Committee.

### 2.2. Stop signal task

Stop signal task was used to assess the ability of motor-inhibitory control (Verbruggen and Logan, 2008). A 13.5-inch Dell laptop running E-Prime v.3.0 (Psychological Software Tools Inc., Pittsburgh, PA, USA) was used to present stimuli and record keypresses. At the beginning, participants were asked to read instructions on the computer screen. On “Go” trials, a black arrow was presented on the screen, and participants were instructed to press the left-arrow key for a leftward pointing arrow with the left index finger, and to press the right-arrow key for a rightward pointing arrow with the right index finger. On “Nogo” trials, a red arrow was presented on the screen, and participants were instructed not to press any key. On “Stop” trials, a “Stop” signal (red arrow) would occur after the “Go” signal (i.e., the black arrow turned red after a delay). Participants were asked to stop their initial response when the “Stop” signal occurred. Participants were instructed to respond as quickly and accurately as possible to black arrows, and not to delay their response to wait in case the “Stop” signal occurred (Ding et al., 2021a).

On each trial, a fixation cross was presented for 300 ms, followed by the “Go” or “Nogo” signal. The maximum response time was set at 1,000 ms, and the intertrial interval was set at 500 ms. On “Stop” trials, the “Stop” signal was presented after the onset of the “Go” signal. At the beginning of the session, the “Stop” signal occurred 250 ms after the “Go” signal. In the trials where response inhibition was successful, the stop signal delay (SSD) was increased by 50 ms on the next “Stop” trial. In the trials where inhibition failed, SSD was decreased by 50 ms on the next “Stop” trial. This ensured an overall successful rate of inhibition [i.e., *P* (respond| signal)] close to 50%. The experiment included 24 practice trials and 400 experimental trials, consisting of 70% “Go” trials, 10% “Nogo” trials, and 20% “Stop” trials, administered in a completely random sequence. The details of SST were described in a previous paper (Ding et al., 2021a).

The SSRT was estimated using the integration method with Go omission replacement (Verbruggen et al., 2019; Ding et al., 2021a), which has been suggested to be more accurate and have higher test-retest reliability than other methods (especially the mean method) for SSRT calculation (Ding et al., 2021a). With the integration method, SSRT was calculated by the mean SSD subtracted from the nth Go reaction time. Here, n stands for a point on the Go reaction time distribution where the integral of the reaction time curve is equivalent to *P* (respond| signal). Go omissions refers to Go trials on which the participants did not respond before the response deadline. In the cases of Go omissions, the SSRT was assigned with the maximum reaction time (RT) (1,000 ms) to compensate for the lack of responses (Verbruggen et al., 2019).

### 2.3. Electroencephalography (EEG)

#### 2.3.1. EEG acquisition

Electroencephalography acquisition was performed after the completion of SST. The participants were seated comfortably in a sound-shielded, dimly lit room for resting-state EEG recording, which lasted 13 min: 6 min with eyes closed, followed by 1 min with eyes open, and 6 min with eyes closed. Scalp EEG signals were recorded using a 128-channel HydroCel Geodesic Sensor Net (Electrical Geodesics, Inc., Eugene, OR, USA) in a geodesic pattern over the surface of the head with a vertex reference. It included 19 contacts at the equivalent 10–20 system locations. The EEG data were digitized and amplified at a 2,000 Hz sampling rate with a Geodesic EEG system 400 (Electrical Geodesics, Inc., Eugene, OR, USA). An online bandpass filter (0.1–100 Hz) was applied and the impedance for the whole net was kept below 10 kΩ throughout data collection (Cai et al., 2021). The 12 min EEG recording with eyes closed was exported after data collection for further analysis.

#### 2.3.2. EEG analysis

Acquired EEG signal were analyzed off-line using MATLAB2019b (Mathworks, Inc., Natick, USA). EEGLAB toolbox (version 14.1.2b) was used for EEG data preprocessing (Delorme and Makeig, 2004). After the EEG data were imported in EEGLAB, the signal was sampled down to 1,000 Hz. Afterward, the EEG data were filtered with a band-pass filter with cut-off values ranging from 0.1 to 40 Hz and segmented in epochs lasting 1,000 ms. The independent component analysis was subsequently performed to exclude components endowing eye (blink and movement), cardiac, and muscular artifacts. The resulting data were further visually inspected to exclude remaining “bad trials” (i.e., amplitudes > 80 μV) and re-referenced using the average signal of every scalp electrode as reference (Cai et al., 2021).

Power and functional connectivity analyses were conducted using customed MATLAB scripts. Absolute power was calculated by fast Fourier transform and averaged in 13–30 Hz for beta band. As we were interested in assessing cortical activities in brain areas including inferior frontal cortex (IFC), motor cortex (MC), and somatosensory cortex (SC), six clusters of electrodes (three clusters for each hemisphere) were selected according to 10–20 system nomenclature (Ding et al., 2022). The averaged power of all electrodes in each cluster was calculated for statistical analysis.

Coherence was calculated using customed MATLAB scripts to reflect functional connectivity between different cortical regions. The Welch’s averaged, modified periodogram method (Welch, 1967), was performed to calculate the squared coherence between each pair of electrodes in four frequency bands. All connectivity matrices were Fisher’s z-transformed (Arun et al., 2020) to the set of Gaussian distributed values and the z-scores were used for further analysis. The averaged z-scores of each pair of electrodes between brain regions of interest were calculated for statistical analysis (Ding et al., 2021b).

GRaph thEoretical Network Analysis (GRETNA) toolbox was used for graph theory analysis (Wang et al., 2015). A graph is based on a set of nodes, and the connections between nodes are edges. Nodes and edges together form the brain network. In the current study, weighted and undirected networks were built based on coherence (Vecchio et al., 2019). As there was no definite method for selecting a single threshold, we integrated the metrics over the entire threshold range (i.e., 0.1–0.4, with an interval of 0.05) to obtain the area under the curve (AUC) to characterize the brain network (Wang et al., 2015; Yan et al., 2017; Ding et al., 2021b). Global efficiency characterizes information transferring ability in the entire brain network (G) (Park et al., 2014). Global efficiency was computed as the average of nodal efficiency across all nodes in the brain network:


(1) $$E_{g\,l\,o\,b\,a\,l}\,\left(G\right)=\frac{1}{N\,\left(N-1\right)}\,\sum_{j\neq i\in G}\frac{1}{D\,\left(i,j\right)}$$

where *D(i, j)* is the shortest path length between node *i* and node *j*, and *N* is the number of nodes in the network.

### 2.4. Statistical analysis

Statistical analysis was performed in Graphpad Prism (version 8.3.0). Data were found to meet the normality assumption using the Kolmogorov–Smirnov test. Pearson correlations were performed to investigate the relationship between physiological data and behavioral data (i.e., SST measures). False discovery rate corrections were applied for multiple correlations. All *P*-values presented in current study are those after the false discovery rate correction. For all analyses, the statistical significance was set at *P* < 0.05.

## 3. Results

### 3.1. Behavioral data

Behavioral data of SST measures are summarized in Table 1.

**TABLE 1:** Behavioral data of SST measures.

Go accuracy (%)	Nogo accuracy (%)	*P* (Go omissions) (%)	Go RT (ms)	RT unsuccessful stop (ms)	*P* (respond| signal) (%)	SSD (ms)	SSRT (ms)
96.88 (3.96)	95.00 (7.30)	1.51 (3.21)	458.92 (97.55)	411.91 (94.34)	46.25 (6.50)	203.81 (126.60)	240.14 (28.76)

Data are presented as mean (standard deviation). SST, stop signal task; RT, reaction time; SSD, stop signal delay; SSRT, stop signal reaction time.

### 3.2. Physiological data

#### 3.2.1. Power analysis

**FIGURE 1:**
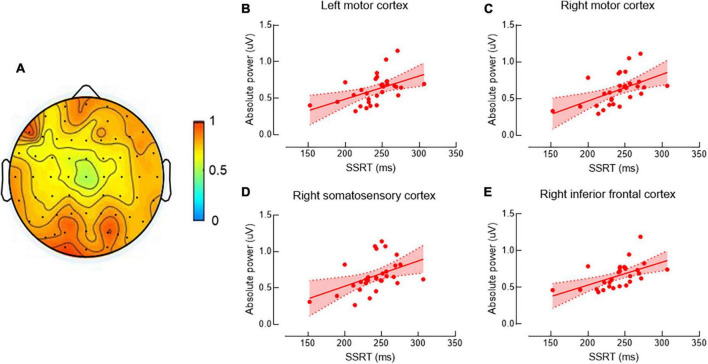
Correlations between beta band power and stop-signal reaction time (SSRT). The topographic map shows beta band power **(A)**. Data are presented as raw values of power. Scatter plots show the significant positive correlations between SSRT and beta band power in left motor cortex (MC) **(B)**, right MC **(C)**, right motor somatosensory cortex (SC) **(D)**, and right inferior frontal cortex (IFC) **(E)**. As longer SSRT is associated with more poor ability of response inhibition, individuals with poor ability of response inhibition tended to have greater electroencephalography (EEG) power in the above brain regions.

Figure 1A shows the beta band power spectrum. Our data revealed significant positive correlations between SSRT and cortical beta power in left and right MC, right SC, and right IFC (*r*’s = 0.49, 0.52, 0.45, and 0.55, *P*’s = 0.031, 0.021, 0.045, and 0.015, respectively) (Figures 1B–E), indicating individuals with poor ability of response inhibition tended to have greater EEG power in those brain regions. There was no significant correlation between other SST measures and cortical beta power in any brain region (*P*’s > 0.05).

#### 3.2.2. Coherence

**FIGURE 2:**
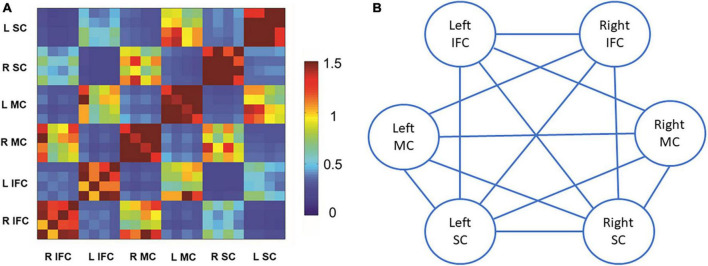
Beta band coherence in each pair of brain regions of interest and the correlations with stop-signal reaction time (SSRT). **(A)** Beta band coherence between bilateral motor cortex, somatosensory cortex, and inferior frontal cortex. Data are presented as z-scores of coherences. There were four electroencephalography (EEG) channels included for each brain region of interest. **(B)** Illustration of correlations between SSRT and beta band coherence in each pair of brain regions of interest. Solid lines stand for significant positive correlations between SSRT and beta band coherence in the pair of brain regions. L stands for left, and R stands for right. SC refers to somatosensory cortex. MC refers to motor cortex. IFC refers to inferior frontal cortex.

**FIGURE 3:**
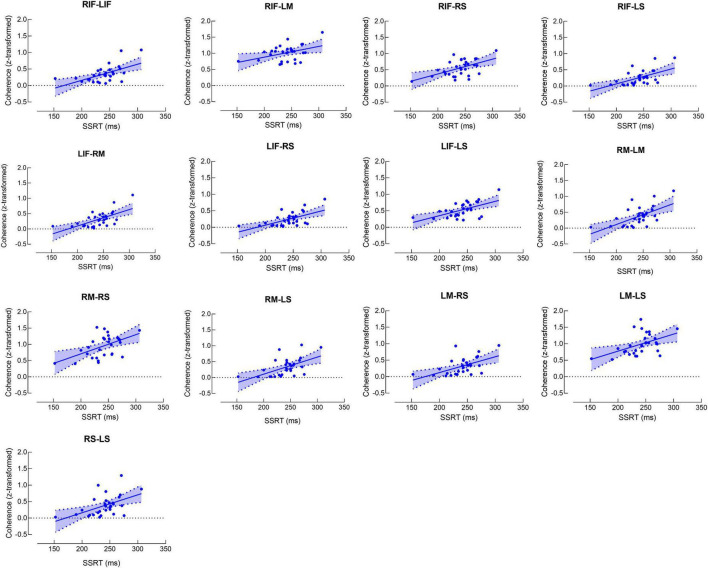
Scatter plots for correlations between stop-signal reaction time (SSRT) and beta band coherence. Each scatter plot represents significant positive correlation between SSRT and beta band coherence in the pair of brain regions. RS and LS refer to right and left somatosensory cortex, respectively. RM and LM refer to right and left motor cortex, respectively. RIF and LIF refer to right and left inferior frontal cortex, respectively.

Figure 2A shows the matrix for beta band coherence between pairs of electrodes in bilateral MC, SC, and IFC. Our data revealed significant correlations between SSRT and coherence of each pair of brain regions (*P*’s < 0.05) (Figures 2B, 3). There was no significant correlation between other SST measures and beta coherence (*P*’s > 0.05).

#### 3.2.3. Graph theory analysis

**FIGURE 4:**
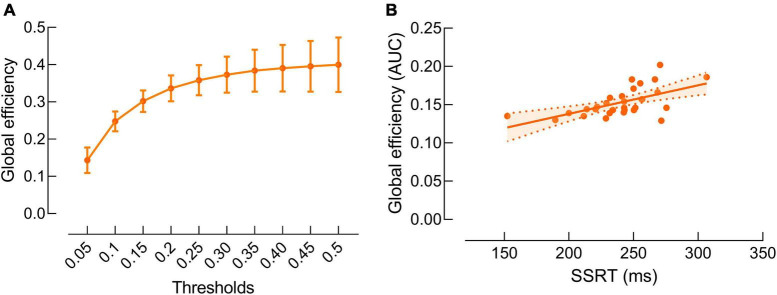
Correlations between beta band global efficiency and stop-signal reaction time (SSRT). **(A)** Beta band global efficiency at each threshold. Data are presented as mean and standard error. **(B)** Significant positive correlation between beta band global efficiency and SSRT. Data are presented as areas under the curves of global efficiency at all thresholds.

Our data revealed significant positive correlation between beta band global efficiency and SSRT (*r* = 0.59, *P* = 0.01) (Figure 4). There was no significant correlation between other SST measures and beta band global efficiency (*P*’s > 0.05).

## 4. Discussion

This study investigated the relationship between resting state cortical beta activity and response inhibition. Our primary findings are (1) beta band EEG power in bilateral sensorimotor cortices and right inferior prefrontal cortex was positively correlated with SSRT; (2) beta band coherence between bilateral sensorimotor and inferior prefrontal cortices was positively correlated with SSRT; (3) beta band global efficiency was positively correlated with SSRT.

### 4.1. Beta band activity

Beta band power in bilateral sensorimotor cortices and right IFC was positively correlated with SSRT, suggesting that individuals with better ability of response inhibition tend to have less resting beta power. To our knowledge, no previous study has investigated the relationship between resting beta power and response inhibition. Prolonged SSRT has been observed in many psychiatric conditions with impaired urge control (i.e., impulsivity), such as attention deficit/hyperactivity disorder (Lijffijt et al., 2005) and schizophrenia (Badcock et al., 2002), suggesting a theoretical link between motor-inhibitory control deficits and impulsivity (Bari and Robbins, 2013; Skippen et al., 2019). Some studies investigated the relationship between resting beta power and trait impulsivity (assessed by questionnaires) and reported that individuals with higher level of trait impulsivity tend to have greater resting beta power (Threadgill and Gable, 2018; Wendel et al., 2021). Our study for the first time investigated the relationship between resting band power and the ability of inhibitory control rather than impulsivity. In line with previous studies (Threadgill and Gable, 2018; Wendel et al., 2021), our results revealed positive correlations between resting beta power and response inhibition and extended the relationship to another aspect of impulsivity.

The neural mechanisms underlying the relationship between beta band oscillatory activity and response inhibition remains unclear. Beta power has been suggested as a signature of an active process promoting the existing motor set whereas compromising neuronal processing of new movements (Androulidakis et al., 2006, 2007; Pogosyan et al., 2009; Engel and Fries, 2010). It has been reported that voluntary movements are slowed if they are triggered during the period of enhanced spontaneous beta band activity (Androulidakis et al., 2006, 2007), suggesting spontaneous enhancement of beta band oscillatory activity relates to impaired motor performance. Previous studies applied transcranial alternating-current stimulation on MC and observed increased resting beta activity accompanied by slowed hand and finger movements (Pogosyan et al., 2009; Wach et al., 2013). Taken together, these evidence suggest that beta band activity possibly signals the tendency of the sensorimotor system to maintain the status quo, and excessive beta band activity would slow down motor performance (Engel and Fries, 2010).

Unlike previous studies investigating the relationship between resting beta activity and velocity of voluntary movement (e.g., visuomotor tracking task) (Pogosyan et al., 2009; Wach et al., 2013), our current study for the first time investigated the relationship between resting beta activity and the ability of response inhibition. Results from previous studies and the current study suggest that excessive beta band activity slows down the velocity of both motor tasks and response inhibition, even though the neural substrates for motor execution and motor inhibitory control are different. This indicates that excessive beta band activity may relate to an overall slowdown of motor performance regardless of the type of movement.

Interestingly, we observed a significant correlation between SSRT and beta band power in bilateral sensorimotor cortex and right IFC, but not in the left IFC. Our results are in line with previous studies reporting that right IFC is an important structure for motor inhibitory control (Aron, 2007; Cunillera et al., 2014, 2016; Chowdhury et al., 2019). As most previous studies (including our current study) included only right-handed participants (Cunillera et al., 2014, 2016), how handedness influences the laterality of motor inhibitory control has not been systematically investigated. Therefore, cautions are needed when generalizing the conclusion that the right IFC, rather than the left IFC, is a critical area in the motor inhibitory network to left-handed individuals.

### 4.2. Beta band functional connectivity

Both beta band global efficiency and coherence between bilateral sensorimotor cortices and IFC were positively correlated with SSRT. Our findings suggest that in addition to beta band oscillatory activity, beta band functional connectivity also associates with response inhibition.

Unlike EEG power reflecting oscillatory synchronization within local cortical neuronal populations, functional connectivity represents neuronal synchronization between distributed cortical regions (Silberstein et al., 2005). Oscillatory synchronization between cortical areas has been increasingly recognized as a critical mechanism in motor organization (Serrien and Brown, 2003; Serrien et al., 2003). Although the relationship between local beta cortical oscillatory activity and impaired motor performance has been extensively investigated (Androulidakis et al., 2006, 2007; Engel and Fries, 2010), fewer studies investigated the relationship between beta band functional connectivity and motor performance (Gilbertson et al., 2005; Silberstein et al., 2005). Silberstein et al. (2005) reported a positive correlation between beta band functional connectivity over distributed cortical regions and motor impairment in PD patients. Gilbertson et al. (2005) reported that the greater beta band corticomuscular coherence was related to the worse motor performance in healthy adults. Although response inhibition is different from other movement type, similar correlations between beta band functional connectivity and motor performance were observed (Gilbertson et al., 2005; Silberstein et al., 2005), suggesting individuals with worse motor performance, regardless of the movement type, tend to have excessive beta band functional connectivity.

We also observed the relationship between beta band global efficiency and the ability of response inhibition. Global efficiency exhibits the efficiency in transporting information at a global scale between genetic brain areas, and greater global efficiency reflects higher information transferring efficiency over the entire brain (Vecchio et al., 2019). Our results suggest that individuals with lower efficiency in transporting information in the brain tend to have worse ability of response inhibition.

### 4.3. Clinical implications

Our current study is the first study to investigate the relationship between resting-state cortical beta band activity and response inhibition. We observed positive correlations between beta band power, coherence and global efficiency and SSRT, indicating individuals with stronger cortical beta band activity tend to have worse ability of inhibitory motor control.

Cortical beta band activity has been reported to be elevated in PD, and PD patients with greater beta band activity tend to have worse motor performance (Silberstein et al., 2005; Jenkinson and Brown, 2011). As PD is characterized as a loss of dopaminergic neurons in basal ganglia, excessive beta band activity has been associated with reduced cortical dopaminergic tone (Jenkinson and Brown, 2011). Silberstein et al. (2005) reported a reduction in cortical beta band activity after dopaminergic therapy accompanied by motor improvement in PD patients, further supporting the existence of a direct relationship between cortical beta activity and dopaminergic tone. Based on this premise, the correlations between beta band activity and response inhibition observed in the current study suggest that dopaminergic neurons possibly play a role in response inhibition.

Despite the extensive existing literature, there is still lack of solid evidence indicating the involvement of dopaminergic neurons in motor inhibitory control (Stinear et al., 2009; Swann et al., 2011; Haynes and Haber, 2013; Benis et al., 2014; Aron et al., 2016; Duque et al., 2017). Suppression an initiated motor output requires both an increase in intracortical inhibition and a reduction in excitatory input from thalamus to primary MC (Duque et al., 2017). Efficient inhibitory control relies on a “hyper-direct” pathway from the frontal cortex to the subthalamus nucleus in basal ganglia, providing a mechanism for rapidly inhibiting the motor system in a global manner (Nambu et al., 2002; Wessel et al., 2016; Wessel and Aron, 2017). As an important neurotransmitter in the cortico-basal ganglia network, dopamine possibly plays a critical role in response inhibition (Lindenbach and Bishop, 2013; Schall et al., 2017). Therefore, the current study provides additional evidence suggesting that dopaminergic neurons are possibly involved in motor inhibitory control.

### 4.4. Limitations

As a pilot study, the sample size of current study is small (*N* = 30). In addition, our sample includes only young adults, which is another limitation of current study. Cautions are needed when generalizing our findings to other populations, such as aging population and PD patients. Future studies are needed to test our results in other populations with larger sample sizes.

## 5. Conclusion

This is the first study to investigate the relationship between resting state cortical beta activity and response inhibition. This study revealed positive correlations between cortical beta band activity, coherence and global efficiency and SSRT, indicating individuals with less cortical beta band activity and functional connectivity tend to have better ability of motor inhibitory control. Our findings have implications on development of new treatment for the diseases with impaired motor inhibitory control.

## Data availability statement

The raw data supporting the conclusions of this article will be made available by the authors, without undue reservation.

## Ethics statement

The studies involving human participants were reviewed and approved by the Guangzhou First People’s Hospital Human Research Ethics Committee. The patients/participants provided their written informed consent to participate in this study.

## Author contributions

YL, HZ, and QD designed the experiment and wrote the manuscript. QD and TL recruited the participants. QD, GC, ZO, and SY conducted the experiments. QD and GC reduced, analyzed, and interpreted the data. All authors contributed to the article and approved the submitted version.
